# Ex vivo biomechanical analysis of flexible versus rigid annuloplasty rings in mitral valves using a novel annular dilation system

**DOI:** 10.1186/s12872-022-02515-x

**Published:** 2022-02-26

**Authors:** Yuanjia Zhu, Annabel M. Imbrie-Moore, Robert J. Wilkerson, Michael J. Paulsen, Matthew H. Park, Y. Joseph Woo

**Affiliations:** 1 Department of Cardiothoracic Surgery. Falk Cardiovascular Research Center, Stanford University School of Medicine, 300 Pasteur Drive, Stanford, CA 94305 USA; 2 Department of Bioengineering, Stanford University, Stanford, CA USA; 3 Department of Mechanical Engineering, Stanford University, Stanford, CA USA

**Keywords:** Mitral annular dilation, Mitral regurgitation, Mitral annuloplasty ring, Biomechanics

## Abstract

**Background:**

Mitral annuloplasty rings restore annular dimensions to increase leaflet coaptation, serving a fundamental component in mitral valve repair. However, biomechanical evaluations of annuloplasty rings are lacking. We aim to biomechanically analyze flexible and rigid annuloplasty rings using an ex vivo mitral annular dilation model.

**Methods:**

Juvenile porcine mitral valves (n = 4) with intercommissural distance of 28 mm were dilated to intercommissural distances of 40 mm using a 3D-printed dilator and were sewn to an elastic mount. Fiber bragg grating sensors were anchored to native chordae to measure chordal forces. The valves were repaired using size 28 rigid and flexible annuloplasty rings in a random order. Hemodynamic data, echocardiography, and chordal force measurements were collected.

**Results:**

Mitral annular dilation resulted in decreased leaflet coaptation height and increased mitral regurgitation fraction. Both the flexible and rigid annuloplasty rings effectively increased leaflet coaptation height compared to that post dilation. Rigid ring annuloplasty repair significantly decreased the mitral regurgitation fraction. Flexible annuloplasty ring repair reduced the chordal rate of change of force (7.1 ± 4.4 N/s versus 8.6 ± 5.9 N/s, *p* = 0.02) and peak force (0.6 ± 0.5 N versus 0.7 ± 0.6 N, *p* = 0.01) compared to that from post dilation. Rigid annuloplasty ring repair was associated with higher chordal rate of change of force (9.8 ± 5.8 N/s, *p* = 0.0001) and peak force (0.7 ± 0.5 N, *p* = 0.01) compared to that after flexible ring annuloplasty repair.

**Conclusions:**

Both rigid and flexible annuloplasty rings are effective in increasing mitral leaflet coaptation height. Although the rigid annuloplasty ring was associated with slightly higher chordal stress compared to that of the flexible annuloplasty ring, it was more effective in mitral regurgitation reduction. This study may help direct the design of an optimal annuloplasty ring to further improve patient outcomes.

## Introduction

Mitral valve regurgitation (MR) is one of the most prevalent causes of global morbidity and mortality [1]. Various etiologies of MR exist. However, physiologically there is a similar pattern where the left atrial dimension increases as the heart compensates for increased regurgitant volume [2]. Therefore, in chronic MR, mitral annular dilation is a common finding, though it seems controversial whether isolated mitral annular dilation is sufficient to cause significant MR [3–5]. Mitral valve annuloplasty rings are a fundamental component in mitral valve repair for MR. The primary purpose of annuloplasty ring implantation is to restore healthy mitral annular dimensions in an effort to increase leaflet coaptation and to prevent future annular dilatation [6–12]. Although a number of mitral valve annuloplasty rings exist with different rigidities, the biomechanical understanding of annuloplasty rings is lacking. Limited information is available regarding the biomechanical and hemodynamic effects after repair using rigid versus flexible mitral annuloplasty rings [13, 14]. Specifically, the chordae rate of change of force or loading rate is an important metric in stress analysis due to the rate dependent stress–strain curves of viscoelastic materials and has been previously shown to correlate well with various repair biomechanics [15]. Currently, ring choice is based on surgeon preference, and clinical studies have demonstrated controversial results. For example, some studies suggested improved left ventricular reconstruction using flexible annuloplasty rings, while others showed superior hemodynamics using rigid annuloplasty rings, or failed to find any differences in clinical outcomes using flexible or rigid rings [16–19]. Therefore, it is prudent to obtain a better understanding of the biomechanical and hemodynamic impact of flexible versus rigid annuloplasty ring on mitral valves.

In order to achieve this, ex vivo heart simulators represent a valuable avenue to quantitatively analyze valvular biomechanics and hemodynamics. We have successfully replicated various mitral valve repair operations using our left heart simulator and have obtained several clinically relevant findings [15, 20–24]. To biomechanically evaluate different mitral annuloplasty rings without the confounding effect from additional mitral valve pathologies, an ex vivo mitral annular dilation model with MR is needed. Previously, we designed a preferential posterior mitral valve annular dilation device [25]. However, the rigidity of this device resulted in an annuloplasty ring repair that was not clinically representative. In this study, we aim to design a novel mitral annular dilation system to compare the biomechanics of repair using flexible or rigid annuloplasty rings in an ex vivo left heart simulator.

## Methods

### Mitral annular dilation system design

**Fig. 1:**
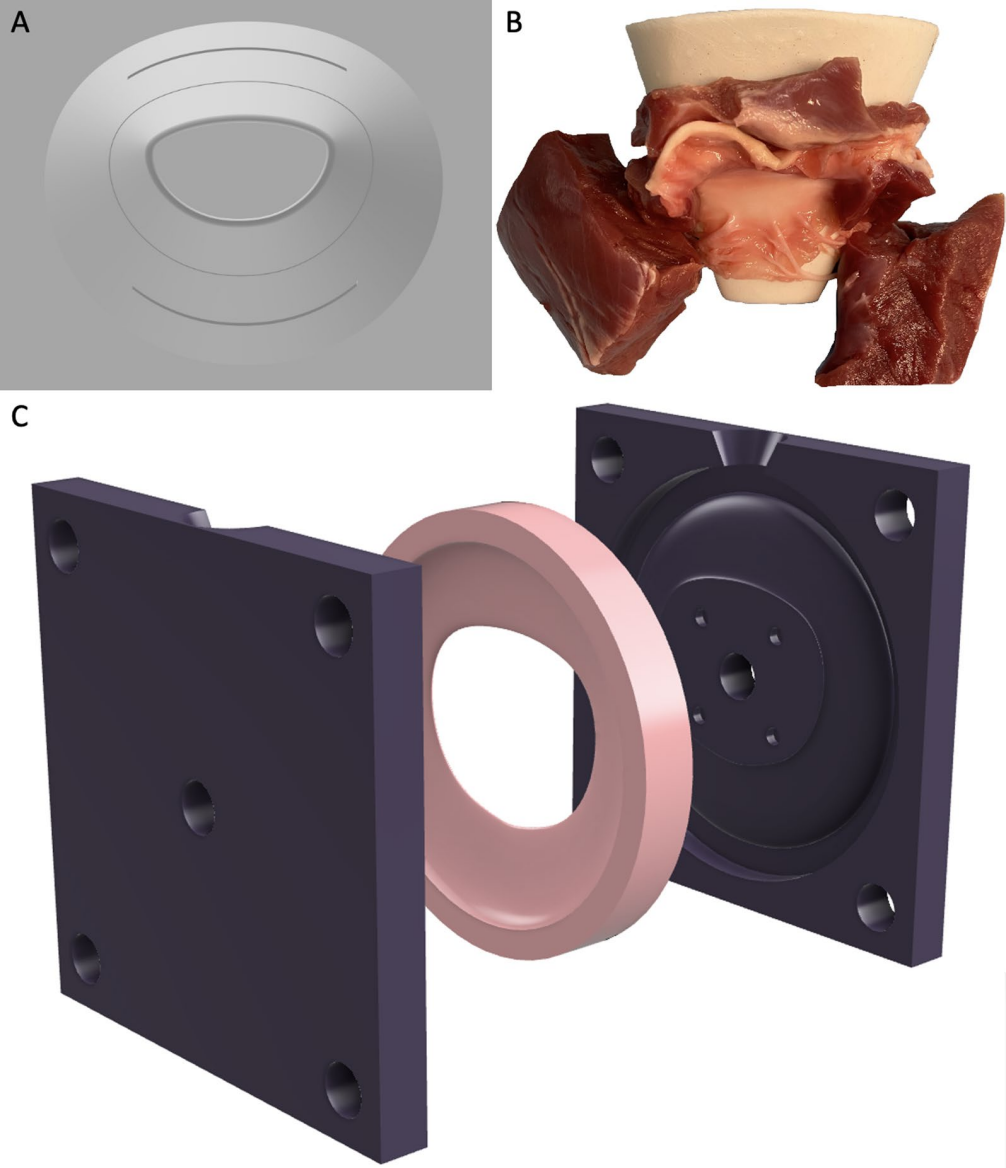
**a** A computer-aided design rendering of the 3D-printed mitral annular dilator. **b** The annular dilation device placed across a juvenile porcine mitral valve to induce annular dilation. **c** Exploded view of the elastic dilated mitral mount produced using 3D-printed molds

The mitral valve annular dilation device was developed using a high-resolution 3D printer (Carbon M2 Printer; Redwood City, CA). To ensure symmetric dilation, the D-shape of a healthy, native mitral annulus was adopted for the device (Fig. 1a). The dilator was designed with the smallest inter-commissure distance of 15 mm, which was gradually increased to the maximal inter-commissure distance of 45 mm. Each mark on the dilator indicates a 10 mm increment of inter-commissure distance. This device was then placed across the explanted mitral valve specimen, and the anterior and posterior annulus was aligned to the correct dilator orientation (Fig. 1b). The dilator was then inserted through the mitral valve, taking care not to injure the chordae tendineae or papillary muscles. Dilation was gradually performed over 6 h until the desired inter-commissure distance was achieved. The dilator was then removed from the valve.

To allow for annular dimension reduction after ring annuloplasty repair without applying excessive force on the left atrial tissue, a flexible mount was needed. This mount must also have a dilated annular dimension to maintain the dilated annular geometry. We designed and 3D-printed a mold that was used to create a highly elastic mount with a dilated annular geometry with an inter-commissural distance to septo-lateral distance ratio of 1:1 using silicone (Silicones, Inc., P-20B Addition Cure silicone, High Point), to allow for proper mounting in the ex vivo left heart simulator (Fig. 1c).

### Sample preparation

Healthy juvenile porcine mitral valves (n = 4) were harvested from hearts obtained from a meat abattoir (Animal Technologies, Tyler, TX). The valve leaflets, annulus, chordae, papillary muscles, and 1 cm of circumferential left atrial tissue were preserved. Only valves with an inter-commissure distance of 28 mm were used in this study to allow for adequate annular dilation to generate MR. Using interrupted 2-0 braided polyester sutures, the undilated valves were mounted to a 3D-printed elastometric polyurethane annular sewing plate that was designed to fit the size of juvenile porcine mitral valves. After baseline data collection, the valves were dilated to inter-commissure distances of 40 mm using the 3D-printed dilation device as described above. The dilated mitral valves were then sewn to the elastic mitral mount using pledgeted interrupted 2-0 braided polyester sutures. After data collection, the valves were repaired using size 28 rigid (Carpentier-Edwards Classic, Edwards Lifesciences) and flexible (Annuloflex, LivaNova) annuloplasty rings in a random order. Data was collected after both annuloplasty ring repairs for each specimen.

### Ex vivo left heart simulator

**Fig. 2:**
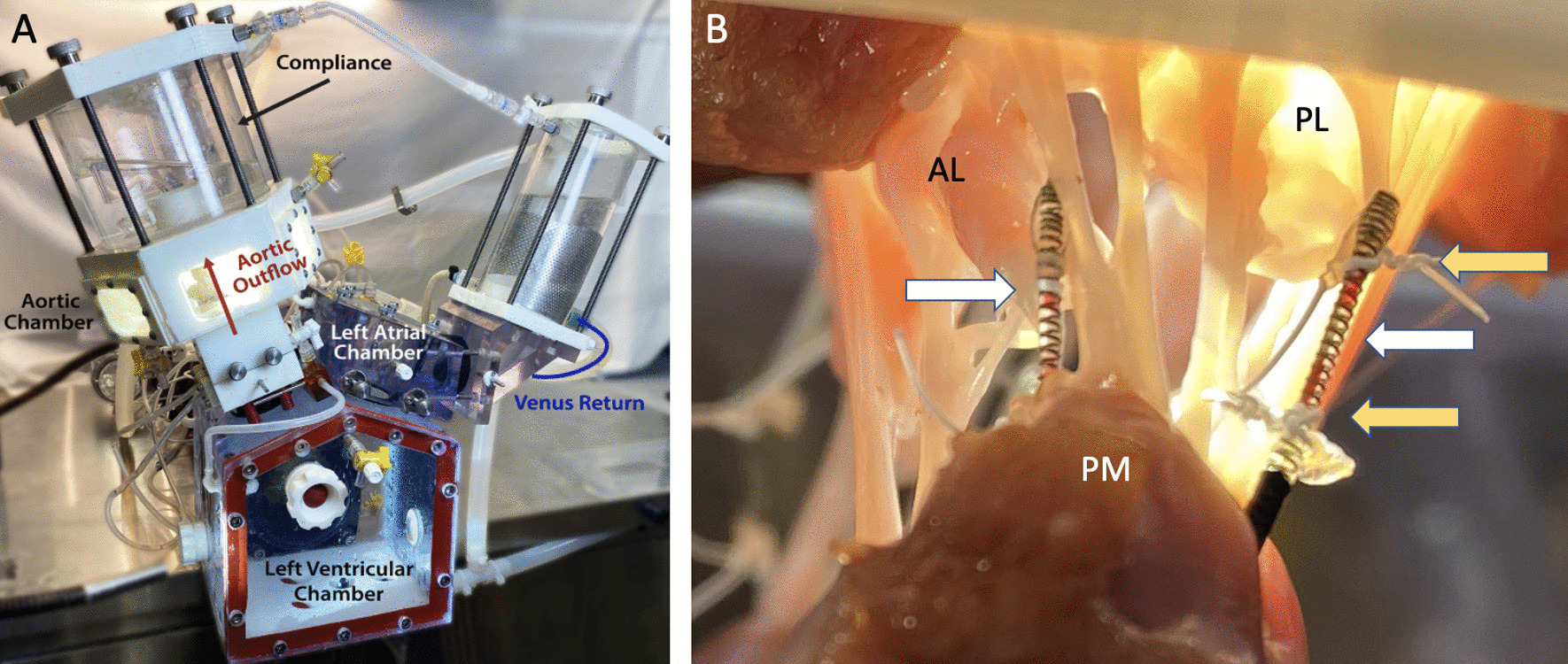
Fiber bragg grating (FBG) sensors denoted by white arrows, instrumented onto mitral valve chordae. Yellow arrows denote polytetrafluoroethylene suture attachment points both proximally and distally on the FBG sensor to the instrumented chordae. *AL* anterior leaflet, *PL* posterior leaflet, *PM* papillary muscle

The left heart simulator, which has been previously described, features a pulsatile linear piston pump (ViVitro Superpump, ViVitro Labs, Victoria, British Columbia, Canada) to generate physiologic parameters using the pump controller and software (ViVitest Software, ViVitro Labs) programmed in accordance with ISO 5840 standards for in vitro valve testing (Fig. 2a) [19–24, 26]. An electromagnetic flow probe (Carolina Medical Electronics, East Bend, NC) was incorporated to record transmitral valvular flow. Ventricular, aortic, and left atrial pressures were measured using pressure transducers (Utah Medical Products, Inc, Midvale, UT). Normal saline was used, and the linear piston pump was programmed to generate a mean arterial pressure of 100 mmHg at 70 bpm. This was achieved by titrating the compliance chambers, peripheral resistance, and pump stroke volume. For each test, hemodynamic data was collected and averaged across 10 complete cardiac cycles. High-speed videography was obtained with an en face view at 1057 frames per second with 1280 × 1024 resolution (Chronos 1.4; Kron Technologies, Burnaby, British Columbia, Canada). Additionally, echocardiogram data was obtained using a Phillips iE33 system with an S5-1 transthoracic probe (Koninklijke Philips NV, Amsterdam, The Netherlands). Coaptation heights were measured using the iE33 on-board software and a Siemens Syngo Dynamics workstation (Siemens Medical Solutions USA, Ann Arbor, MI).

### Chordae tendineae force measurement

Chordae tendineae force data were collected after dilation and ring annuloplasty repairs using the flexible and rigid rings. This was achieved by implanting previously described, calibrated, high-resolution fiber bragg grating (FBG) sensor (DTG-LBL-1550, 125 μm; FBGS International, Geel, Belgium) (Fig. 2b) [20, 26, 27]. These sensors were attached to the native chordae using CV-5 polytetrafluoroethylene sutures flanking each side of the strain gauge. The chordae were then cut between the two suture attachment sites so that force on the chordae was imparted to the FBG sensors. Due to the variation in chord geometry from valve to valve, this was completed for both the anterior and posterior chordae, and at least 4 chordae were instrumented for each valve. Forces measured from all chordae were averaged. Peak chordal forces over a cardiac cycle and the rate of change of force after normalization to transmitral mean pressure in systole were analyzed in MATLAB (MathWorks, Natick, MA).

### Statistical analysis

Continuous variables were reported as mean ± standard deviation unless specified otherwise. A 2-sampled paired t test was performed to compare baseline versus post-dilation, post-dilation versus post-flexible annuloplasty ring repair, post-dilation versus post-rigid annuloplasty ring repair, and post-flexible versus rigid annuloplasty ring repair in terms of hemodynamic and/or chordal force data. Statistical significance was defined at *p* < 0.05 for all tests.

## Results

**Fig. 3:**
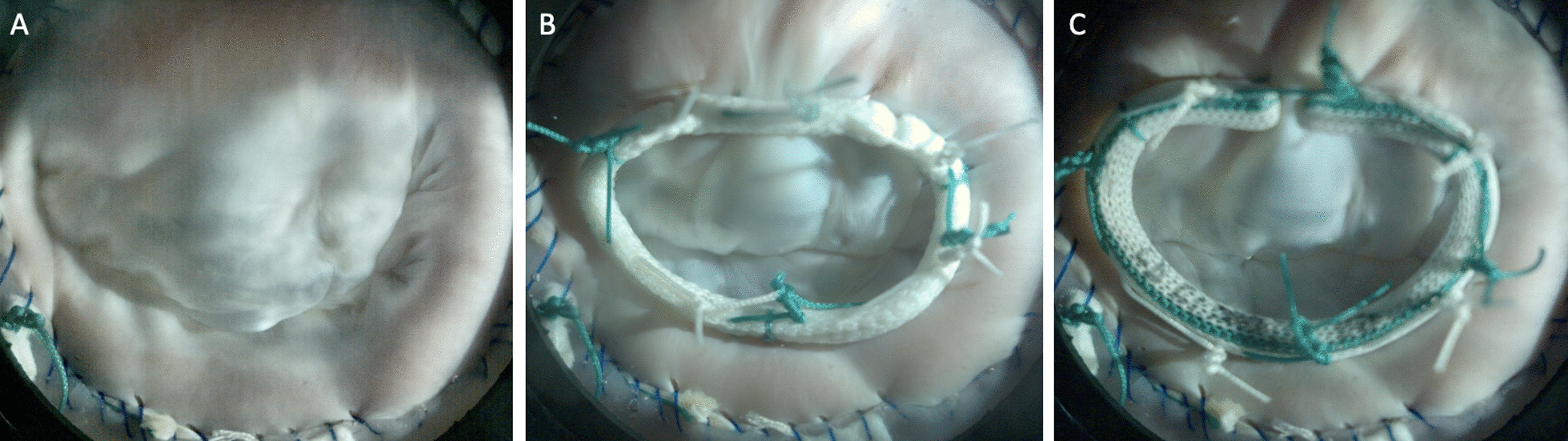
**a** En face view of an example juvenile mitral valve after annular dilation, followed by ring annuloplasty repair using a **b** flexible and a **c** rigid annuloplasty ring

**Fig. 4:**
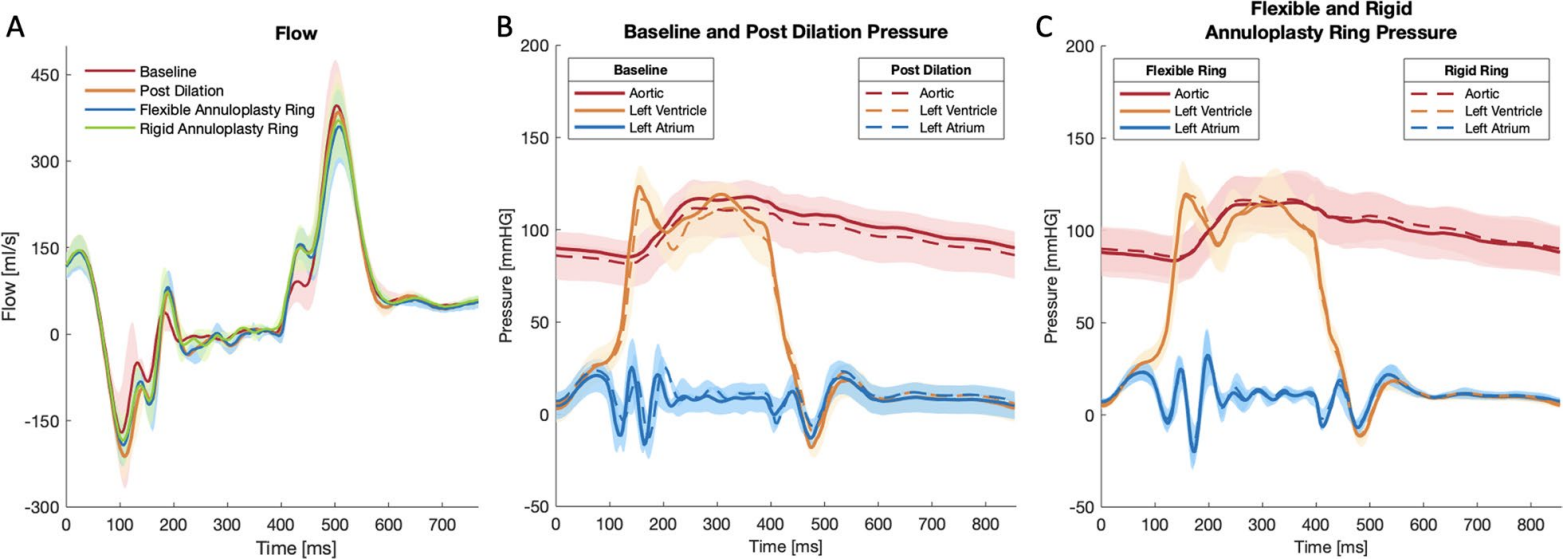
**a** Mean transmitral flow tracings confirmed mitral regurgitation after annular dilation. Rigid annuloplasty ring repair successfully eliminated mitral regurgitation. **b**, **c** Mean pressure tracings at baseline, post dilation, and after ring annuloplasty repair. The shaded areas represent standard deviation

The mitral annular dilation system successfully generated an ex vivo mitral annular dilation model with MR (Fig. 3a). As evidenced by the en face view of an example of a dilated porcine mitral valve, there was inadequate leaflet coaptation in systole captured by the high-speed videography. The leaflet coaptation height measured by echocardiography post dilation decreased to 1.0 ± 0.2 cm from 1.5 ± 0.3 cm at baseline (*p* = 0.05). The mitral regurgitation fraction post dilation increased to 23.4 ± 5.2% from 13.9 ± 4.4% at baseline (*p* = 0.14). MR was also observed from the mean mitral flow tracings, as evidenced by the flow reversal observed in systole in the post dilation state compared to baseline (Fig. 4a). Mean aortic, ventricular, and left atrial pressure tracings are shown in Fig. 4b. Mean left atrial and ventricular pressure measured for baseline versus post dilation were 8.6 ± 7.8 mmHg and 43.6 ± 5.5 mmHg versus 11.1 ± 0.8 mmHg and 43.3 ± 5.7 mmHg, respectively. A summary of hemodynamic data is shown in Table 1.

**Table 1:** Hemodynamic parameters measured at baseline, post dilation, and after ring annuloplasty repair using flexible and rigid rings

Hemodynamic parameters	Baseline	Post dilation	Flexible annuloplasty ring	Rigid annuloplasty ring	P value baseline versus post dilation	P value post dilation versus flexible annuloplasty ring	P value post dilation versus rigid annuloplasty ring
Heart rate (bpm)	70 ± 0	70 ± 0	70 ± 0	70 ± 0	–	–	–
Pump stroke volume (mL)	101.0 ± 0.1	109.9 ± 0.1	109.9 ± 0.0	109.9 ± 0.1	0.87	0.81	0.61
Effective stroke volume (mL)	51.2 ± 6.3	46.1 ± 10.1	46.0 ± 10.0	50.5 ± 7.4	0.09	0.97	0.06
Cardiac output (L/min)	3.6 ± 0.4	3.2 ± 0.7	3.2 ± 0.7	3.5 ± 0.5	0.09	0.97	0.06
Mean aortic pressure (mmHg)	101.3 ± 2.6	96.7 ± 14.0	99.0 ± 14.1	101.0 ± 13.0	0.53	0.05	**0.03**
Systolic aortic pressure (mmHg)	118.5 ± 2.7	112.8 ± 15.5	115.3 ± 15.7	117.9 ± 13.6	0.48	0.18	**0.04**
Diastolic aortic pressure (mmHg)	85.1 ± 3.0	81.5 ± 12.7	83.4 ± 12.8	85.2 ± 12.1	0.60	0.07	**0.03**
Ventricular mean pressure (mmHg)	43.6 ± 5.5	43.3 ± 5.7	44.3 ± 6.4	45.0 ± 6.5	0.94	0.15	**0.04**
Atrial mean pressure (mmHg)	8.6 ± 7.8	11.1 ± 0.8	11.0 ± 2.2	11.3 ± 2.4	0.55	0.89	0.86
Mitral valve mean gradient (mmHg)	1.1 ± 1.3	0.8 ± 0.4	1.7 ± 0.4	1.3 ± 0.3	0.74	**0.004**	0.21
Mitral forward flow time (s)	0.6 ± 0.0	0.5 ± 0.0	0.6 ± 0.0	0.6 ± 0.0	0.18	0.08	0.12
Mitral forward volume (mL)	59.9 ± 10.0	59.6 ± 9.5	58.3 ± 9.3	61.4 ± 8.2	0.78	0.52	0.13
Mitral closing volume (mL)	− 7.2 ± 4.8	− 11.8 ± 1.9	− 10.7 ± 0.6	− 10.5 ± 1.3	0.17	0.25	0.06
Mitral regurgitant fraction (%)	13.9 ± 4.4	23.2 ± 5.5	21.6 ± 6.1	17.8 ± 2.8	0.14	0.32	**0.05**
Transmitral forward energy loss (mJ)	− 2.8 ± 16.2	− 14.0 ± 7.3	− 5.5 ± 10.1	− 11.0 ± 7.7	0.12	**0.04**	0.64
Transmitral closing energy loss (mJ)	31.3 ± 27.3	51.4 ± 8.7	58.1 ± 14.4	56.6 ± 14.8	0.19	0.54	0.58
Transmitral leakage energy loss (mJ)	20.1 ± 11.0	25.3 ± 9.1	21.3 ± 25.8	7.7 ± 16.5	**0.02**	0.81	0.24
Transmitral total energy loss (mJ)	48.6 ± 7.0	62.7 ± 8.5	73.9 ± 22.6	53.4 ± 11.2	0.13	0.34	0.28

Data presented as mean ± standard deviation. Values in bold designate statistical significance

After ring annuloplasty repair, mitral valve competency was restored to a varied degree. As shown in Fig. 3b, c, proper leaflet coaptation was reinstated with both the flexible and the rigid annuloplasty ring. Similarly, leaflet coaptation height measured by echocardiography was significantly increased after ring annuloplasty repair using the flexible versus the rigid annuloplasty ring compared to that of the post dilation state (*p* = 0.05 versus *p* = 0.03). Specifically, the coaptation height after repair using the flexible versus the rigid annuloplasty ring was 1.2 ± 0.2 cm versus 1.2 ± 0.1 cm (*p* = 0.15), respectively. The mitral regurgitation fraction also decreased after ring annuloplasty repair using the flexible (21.6 ± 6.1%) and the rigid annuloplasty ring (17.8 ± 2.8%). Compared to post dilation, the reduction in the mitral regurgitation fraction was significant using the rigid annuloplasty ring (*p* = 0.05). There was no difference in mitral regurgitation fraction after flexible (*p* = 0.23) or rigid ring repair (*p* = 0.33) compared to baseline prior to annular dilation. The elimination of MR using the rigid annuloplasty ring was also observed from the mean mitral flow tracings, as the flow tracing returned to baseline (Fig. 4a). However, residual MR was observed after repair using the flexible annuloplasty ring with continued flow reversal in systole (Fig. 4a). Mean aortic, ventricular, and left atrial pressure tracings after ring annuloplasty repair are also shown in Fig. 4c. Mean left atrial and ventricular pressure measured after ring annuloplasty repair using the flexible versus rigid ring were 11.0 ± 2.2 mmHg and 44.3 ± 6.4 mmHg versus 11.3 ± 2.4 mmHg and 45.0 ± 6.5 mmHg, respectively.

**Fig. 5:**
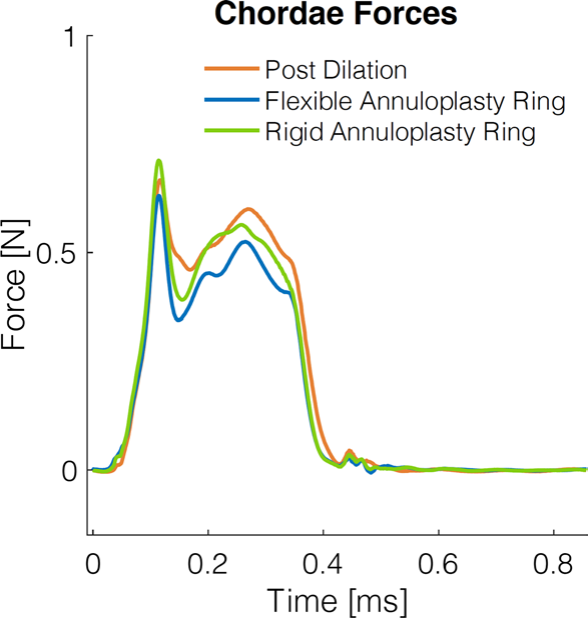
Composite chordal force tracings over the course of a complete cardiac cycle measured at post dilation and after ring annuloplasty repair

By restoring leaflet coaptation and reducing MR, flexible ring annuloplasty repair resulted in a significantly lowered rate of change of force on chordae (Table 2). Flexible compared to rigid annuloplasty ring repair was associated with reduced chordal rate of change of force 7.1 ± 4.4 N/s versus 9.8 ± 5.8 N/s (*p* = 0.0001). Additionally, flexible annuloplasty ring repair significantly reduced peak chordal forces (0.6 ± 0.5 N) compared to the post dilation state (0.7 ± 0.6 N, *p* = 0.01). Similarly, the peak chordal forces were found to be higher after rigid annuloplasty ring repair (0.7 ± 0.5 N) compared to after flexible annuloplasty ring repair (*p* = 0.01). Composite force tracings are shown in Fig. 5.

**Table 2:** Chordal forces post dilation and after ring annuloplasty repair using flexible and rigid rings

Chordal forces	Post dilation	Flexible annuloplasty ring	Rigid annuloplasty ring	P value post dilation versus flexible annuloplasty ring	P value post dilation versus rigid annuloplasty ring	P value flexible versus rigid annuloplasty ring
Rate of change of force (N/s)	8.6 ± 5.9	7.1 ± 3.4	9.8 ± 5.8	**0.02**	**0.01**	**0.0001**
Peak force (N)	0.7 ± 0.6	0.6 ± 0.5	0.7 ± 0.5	**0.01**	0.35	**0.01**

Data presented as mean ± standard deviation. Values in bold designate statistical significance

## Discussion

The mitral annular dilation system successfully generated an ex vivo mitral annular dilation model. Through symmetrical annular dilation, leaflet coaptation was reduced, leading to MR. It was also crucial to ensure that the elastic mitral mount designed for this study allowed for maximal annular dimension restoration via ring annuloplasty repair. Using the ex vivo left heart simulator, we showed that both the flexible and rigid annuloplasty rings were effective in increasing mitral leaflet coaptation height. Additionally, annuloplasty ring repair further reduced chordae rates of change of force from the diseased, post dilation state. Although rigid annuloplasty rings were found to be associated with increased peak chordal forces compared to that of the post dilation state, they were more effective in reducing MR compared to that after  flexible ring annuloplasty repair.

In this study, the mitral annulus was effectively dilated using our dilation system, and MR was successfully induced. Clinically, left ventricular dysfunction, leaflet tethering, and degenerative changes to the mitral valve apparatus are important features found in patients with significant MR [2, 28, 29]. These pathologic changes further exhaust physiologic leaflet coaptation area. Nonetheless, annular dilation is a ubiquitous finding in patients with MR due to the chronic left atrial adaptation to increased regurgitant volume [2]. As we aim to perform biomechanical analysis of mitral annuloplasty rings, whose main function is to restore mitral annular dimensions, our isolated mitral annular dilation model is the ideal ex vivo system to carry out the experiment. This allows us to eliminate potential confounding factors that may be associated with other mitral valve repair techniques that must be employed to achieve mitral valve competency in addition to ring annuloplasty repair.

We found that the rigid annuloplasty ring was more effective in reducing MR in our model. The rigid rings, in contrast to the flexible rings, were designed to restore the mitral annulus to its normal size and shape to increase leaflet coaptation without allowing annular changes during the cardiac cycle. The rigidity of the annuloplasty ring may play a crucial role in MR recurrence based on clinical observations after repair using flexible rings [17]. Thus the shape of the mitral annulus, in addition to reduction of its area after annuloplasty, might play an important role in MR elimination. However, keeping the otherwise dynamic mitral annulus in a fixed geometry may come with drawbacks. We showed that flexible annuloplasty rings were associated with lower chordal forces compared to that measured in the post dilation state, and it has been theorized that when the optimal coaptation surface is disrupted, as in the case with mitral valves with dilated annulus, the chordae are forced to take on additional forces [30]. After the leaflet coaptation surface is restored with ring annuloplasty repair, chordal forces are expected to decrease [20, 21]. In this study, the peak chordal force did not change after rigid annuloplasty ring repair, but a higher chordal rate of change of force compared to post dilation was observed. We also showed that the rigid annuloplasty ring was associated with higher chordal stresses compared to those associated with the flexible annuloplasty ring. Specifically, the rate of change of force is an important metric. In viscoelastic materials, such as most biologic tissues, the mechanical properties of the tissue changes as the rate of loading changes [15]. The increase in rate of change of force, therefore, may have a significant impact on repair durability. We hypothesize that the plasticity within the flexible ring, compared to the rigid ring, allowed for a more gradual change of force and an improved chordal force dampening effect when the leaflets close as the left ventricle starts to contract to impose increased force onto the chordae. Furthermore, previous studies demonstrated that the absorbed forces were lower in semi-rigid rings compared to flexible rings, suggesting possibly improved myocardial stress adaptation and decreased risk of ring dehiscence and repair failure [31, 32]. An optimal mitral annuloplasty ring that demonstrates selective rigidity and flexibility may be able to further improve repair durability.

One area of refinement for this model is to better mimic the in vivo mitral annular dynamic changes throughout a cardiac cycle, as our current model simulates a fixed, planar annular geometry. Additionally, more precise replication of specific mitral annular geometries associated with various MR pathologies can be pursued. For example, ischemic MR is commonly associated with asymmetric annular dilation [34]. Being able to simulate different annular dilation geometries can enable us to further assess the impact of different annuloplasty rings and other repair techniques. Furthermore, our model simulated an acute change in mitral annular dimension, whereas clinically, mitral annular dilation progresses over time. Our model was unable to capture the chronic adaptation that typically happens to the mitral valve apparatus as observed in patients with chronic MR [34]. It would also be interesting to evaluate forces on the papillary muscles after annuloplasty ring repair [35]. Further validation of the results found in this study can be performed using previously published force models on mitral valves. Lastly, to further improve the understanding of rigid and flexible annuloplasty rings’ effect on mitral annular geometry, detailed analysis of septo-lateral geometry throughout a cardiac cycle should be performed with an ex vivo system that more accurately mimics the tissue property to create an appropriate degree of resistance to annular dimension reduction from annuloplasty.

## Conclusions

In conclusion, our dilation system was successful in generating an isolated mitral annular dilation model with associated MR. Both the flexible and rigid annuloplasty rings were effective in increasing leaflet coaptation height. Although the rigid annuloplasty ring was associated with higher chordal stresses compared to those of the flexible annuloplasty ring, it was more effective in reducing MR. This study provides important biomechanical evidence for the use of flexible and rigid annuloplasty rings to restore proper mitral annular dimensions and may help direct the design of an optimal annuloplasty ring to further improve patient outcomes.

## Data Availability

The datasets used and/or analysed during the current study are available from the corresponding author on reasonable request.

## Abbreviations

AL: Anterior leaflet
FBG: Fiber bragg grating
MR: Mitral valve regurgitation
PL: Posterior leaflet
PM: Papillary muscle
